# Acute effects of Tai Chi with different practice emphasis on autonomic activity

**DOI:** 10.3389/fspor.2025.1642123

**Published:** 2025-09-01

**Authors:** Dejian Duan, Haojie Huang, Wenbo Li, Cong Xiao, Dong Wang

**Affiliations:** ^1^ Department of Physical Education, Xiamen University, Xiamen, China; ^2^ Center for Sports and Health Promotion Research and Communication, Xiamen University, Xiamen, China; ^3^ Department of Physical Education, China University of Geosciences, Beijing, China; ^4^ School of Rehabilitation, Gannan Medical University, Ganzhou, China; ^5^ Wushu and Dance School, Shenyang Sports University, Shenyang, China

**Keywords:** Tai Chi, heart rate variability, mind, autonomic nerves, health

## Abstract

**Introduction:**

Exercise intensity, breathing patterns, and intentional meditation significantly influence autonomic nervous system activity. Tai Chi serves as an aerobic exercise stimulus. Additionally, it incorporates a distinctive breathing pattern. It is also seen as a meditative exercise. However, the acute effect of different Tai Chi factors on autonomic activity is unclear.

**Objective:**

This study aims to investigate the acute effects of Tai Chi with different practice emphasis on autonomic activity. The findings may guide the selection of Tai Chi techniques in exercise prescription.

**Methods:**

After health screening, 8 Traditional Chinese Medicine students (TCMS), 8 Competitive Tai Chi students (CTCS), and 9 Economics and Management students (EMS) were enrolled. Participants were assessed for indices related to autonomic activity, exercise intensity, respiration, and mind state during both quiet and Tai Chi conditions.

**Results:**

Results indicated that the RMSSD and SDNN values for TCMS were significantly higher than those of CTCS and EMS (*P* < 0.05). Heart rate was not significantly different between the groups during quiet and Tai Chi states (*P* > 0.05). Immediately following Tai Chi practice, TCMS exhibited significant reductions in thoughtfulness, anger, and inactivity (*P* < 0.05). No significant differences were noted in CTCS and EMS (*P* > 0.05).

**Conclusion:**

Tai Chi practices emphasizing mindfulness may be more effective in enhancing autonomic activity. It recommends the standing poles interventions for depression, insomnia, and other groups for more autonomic health benefits.

## Introduction

The autonomic nervous system comprises sympathetic and parasympathetic pathways that continuously collaborate to maintain metabolic homeostasis (1). High autonomic activity in the quiet state and higher parasympathetic activity than sympathetic activity means that the internal organs are functioning well and the body is healthy (2). Conversely, diminished autonomic activity during extended periods of rest, coupled with increased sympathetic relative to parasympathetic activity, suggests metabolic dysfunction and poor health status (3). The strength of autonomic activity is an important indicator of the functional state of the organism (4). Research has established a correlation between reduced autonomic activity and the onset of chronic conditions (5), including diabetes (6), coronary heart disease (7), and heart failure (8). A review of the literature identifies exercise intensity, respiratory patterns, and emotional states as primary determinants of autonomic activity (9).

Firstly, aerobic exercise has been shown to enhance autonomic function (10). Exercise intensity is a key factor influencing autonomic activity (11). The intensity of exercise enhances autonomic nervous system function by stimulating both internal and external receptors, modulating fluid secretion in the central nervous system, and inducing adaptive changes in autonomic activity (12). Increased exercise intensity activates pressure and chemoreceptors in internal organs, transmitting nerve impulses to higher brain centers (13). Central nervous system activation leads to heightened norepinephrine secretion from sympathetic ganglia and increased sympathetic nerve activity, alongside reduced acetylcholine secretion from parasympathetic ganglia and diminished parasympathetic activity. The cardiac sinus node is innervated by both sympathetic and parasympathetic nerves (14). CNS alterations result in cardiac sympathetic excitation, vagal inhibition, elevated heart rate, and a reduction in the RR interval (15). Long-term aerobic exercise improves the autonomic coordination-antagonism relationship, the overall level of functioning increases, and a new equilibrium is achieved at a higher level of functioning (16).

Secondly, sinus arrhythmia induced by breathing patterns can further stimulate autonomic activity (17). One perspective posits that respiratory centers modulate preganglionic neurons (18). During inspiration, sympathetic activity increases, resulting in an elevated heart rate. Conversely, during expiration, parasympathetic activity rises, leading to a decrease in heart rate. Concurrently, pressure receptors in the lungs relay information to the respiratory center, facilitating real-time regulation of cardiovascular activity (19). An alternative perspective is the localized reflex theory (20). This theory suggests that respiratory-induced sinus arrhythmia is mediated by fluctuations in intrathoracic pressure. Respiratory muscles facilitate the expansion and contraction of lung tissues, resulting in alterations in alveolar volume and air pressure (21). During inspiration, alveolar volume increases, pressure decreases, alveolar capillaries dilate, and blood volume within the alveoli rises. The augmented alveolar blood volume leads to reduced pulmonary venous return, diminished left ventricular output per contraction, and lowered arterial blood pressure (22). Stimulation of arterial pressure receptors triggers sympathetic excitation, vasoconstriction, and an increase in heart rate (23). The expiratory phase is the opposite of inhalation, with parasympathetic excitation, vasodilatation, and decreased heart rate. Both pathways evidently influence autonomic activity. Breathing patterns can induce acute alterations in central cardiovascular activity and localized cardiovascular stress, thereby affecting autonomic activity (24). Sustained exercise optimizes breathing patterns, enhances central regulation, and improves feedback from pulmonary pressure receptors, ultimately increasing autonomic activity.

Lastly, emotional activity is intricately linked to autonomic function (25). Neurotransmitters (such as dopamine and serotonin), hormonal secretions (including testosterone and cortisol), and structural and functional brain remodeling underpin variations in emotional states (26). External stimuli induce mood alterations by activating the central nervous system, leading to modifications in signaling molecules and autonomic nervous system responses (27). Consequently, engaging in positive thinking meditation influences autonomic activity, promoting psychospiritual relaxation and mitigating anxiety and depression-related emotions (28).

Factors related to exercise intensity, breathing patterns, and meditation significantly impact autonomic nerve function. Tai Chi integrates components of exercise intensity, distinctive breathing patterns, and focused meditation (29). Numerous studies indicate that Tai Chi practice markedly enhances autonomic activity, alleviates insomnia, and mitigates depressive symptoms (30). However, the relative impact of acute effects on autonomic activity from exercise intensity, breathing patterns, and meditative components of Tai Chi exercise is unclear.

Distinct populations practicing Tai Chi emphasize varying technical focuses. Beginners primarily concentrate on mastering the movements. At that stage, beginners mainly practice programmed Tai Chi routines. For these individuals, Tai Chi serves predominantly as an aerobic exercise. The health benefits derived from Tai Chi for beginners primarily stem from the effects of exercise intensity. Competitively trained practitioners emphasize synchronizing breath with movement. Professional athletes prioritize training in the distinctive breathing patterns inherent to Tai Chi. They preach an open movement pattern of inhaling and a closed movement pattern of exhaling. Mastery of these unique breathing patterns enhances their athletic performance in competitive settings. Proficient training in deep, prolonged, slow, and even breathing patterns is essential for all Tai Chi athletes. Breathing patterns constitute a critical factor for success in competitive Tai Chi. Practitioners specializing in Chinese medicine predominantly emphasize Tai Chi meditation. The medical principles underlying traditional Tai Chi are largely derived from Chinese medicine theory. These practitioners enhance their comprehension of Chinese medical theories through Tai Chi practice. They prioritize intentional training, often through static poles, to cultivate awareness of bodily sensations and promote relaxation of both body and mind. Thus, the intensity of movement is a notable characteristic of Tai Chi practice among beginners. Breathing patterns serve as a distinguishing characteristic of Tai Chi practice among competitive athletes. Meditation represents a defining aspect of Tai Chi practice for those specializing in Chinese medicine.

Therefore, the aim of this study was to explore the acute effects of different factors of Tai Chi on autonomic nerves through a population with different Tai Chi practice characteristics. This research will enhance the understanding of Tai Chi-specific characteristics and serve as a reference for selecting Tai Chi techniques and optimizing exercise prescriptions in the future.

## Methods

### Participants

All participants provided written informed consent and were informed of the experimental procedures and objectives. All study methods adhered to the relevant guidelines of the Declaration of Helsinki and received ethical approval from Beijing Sport University (NO.2023161H). Informed consent was obtained from all subjects. Sample size reference previous studies (50).

A total of 36 healthy male college students with prior Tai Chi experience were recruited for this study. Following screening, 25 participants were included for analysis. The cohort comprised 8 students majoring in competitive Tai Chi, 8 in traditional Chinese medicine, and 9 in economics and management, as detailed in Table 1.

**Table 1:** List of basic information of Tai Chi practitioners.

Group	Age (years)	Height (cm)	Weight (kg)	BMI (kg/m^2^)
Competitive Tai Chi students (*n* = 8)	21 ± 2	176 ± 3	68 ± 8	22 ± 3
Traditional Chinese medicine students (*n* = 8)	22 ± 1	174 ± 5	68 ± 8	23 ± 3
Economics and management students (*n* = 9)	19 ± 1	177 ± 5	72 ± 20	23 ± 6

#### Competitive Tai Chi students

They are athletes specializing in Tai Chi at the China Wushu School of Beijing Sport University. These practitioners possess over 6 years of Tai Chi training and have achieved at least a national level 2 athlete status. All participants were recognized for their technical proficiency and recommended by three qualified National Wushu Routine Level 1 judges. Their primary practice involves Tai Chi competition routines, with a strong emphasis on the coordination of movement and breathing. They primarily practice the prescribed competition routines of major Tai Chi styles, including the 56-form Chen style, 40-form Yang style, 46-form Wu (Hao) style, 45-form Wu style, 73-form Sun style, and the integrated 42-form. Additionally, they practice self-choreographed, high-difficulty Tai Chi routines designed for competitive performance.

#### Traditional Chinese medicine students

They are long-time Tai Chi practitioners at the Medicine and Wu Shu Union (Tai Chi club) of the Beijing University of Chinese Medicine. These practitioners lack formal athletic training backgrounds and are college students majoring in Chinese medicine-related fields, having practiced traditional Tai Chi for 2–3 years. They practice Tai Chi five or more times weekly for one hour per session, totaling over five hours per week under the guidance of a traditional master. Their primary exercises include standing poles (Zhan Zhuang) and traditional Chen-style Tai Chi. They place particular emphasis on intentional meditation to enhance physical fitness and inner awareness.

#### Economics and management students

They are college students taking a general Tai Chi class at the School of Management at Beijing Sport University. These practitioners have no prior athletic training and possess only one semester of Tai Chi experience, totaling 64 credit hours. They attend Tai Chi classes twice weekly for 1.5 h per session, achieving attendance rates that meet examination standards and demonstrating proficiency in technical movements. Their primary exercises include 24-Form Tai Chi and 16-Form Tai Chi Sword, focusing on mastering movements and routines.

Inclusion criteria consisted of the following: (1) Physical Activity Readiness Questionnaire, PAR-Q was answered “no” to all seven questions. (2) The electrocardiogram was unremarkable. (3) They had not eaten in the hour before the test and were emotionally stable. (4) They slept 6 h or more the night before the test.

Exclusion criteria were defined as follows: (1) They have a chronic illness or a history of significant illness. (2) They have experienced sports injuries and fractures in the last three years. (3) They had heavy physical activity (e.g., basketball, running, skiing, etc.) in the 72 h prior to the test. (4) They had smoked, drank, or stayed up late in the 24 h before the test. (5) Subjects had taken coffee or stimulant drugs within 24 h prior to testing.

### Experimental procedure

**Figure 1:**
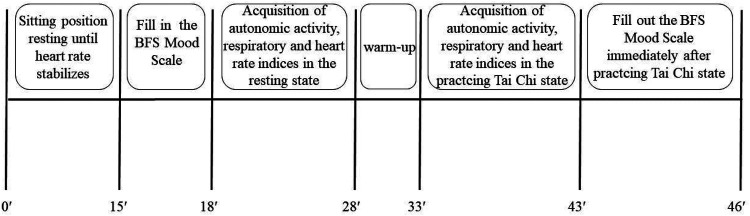
Overall flow chart of the experiment.

On the test day, subjects rested for approximately 15 min before completing the BFS Mood Scale and donning the data collection device once their heart rates stabilized. Following this, data on autonomic activity, intensity, and respiratory parameters were collected during a 10-min seated quiet state. Subsequently, subjects engaged in a 5-min warm-up followed by a 10-min Tai Chi practice. Data on autonomic activity, intensity, and respiration were recorded throughout the Tai Chi practice. The BFS Mood Scale was administered immediately following the Tai Chi practice. The overall experimental flow is illustrated in Figure 1. Test operators possessed experience in conducting cardiorespiratory endurance tests for Chinese national team athletes and were proficient in instrument operation and emergency management. Testing was conducted at the professional martial arts gym at Beijing Sport University.

### Tai Chi exercise protocol

Tai Chi exercises were selected from two classic movements: “Ye Ma Fen Zong” and “Dao Juan Gong”. Subjects performed five forward movements of “Ye Ma Fen Zong” and five backward movements of “Dao Juan Gong”, repeating the sequence for 10 min. The initial movement served as the starting point for data collection, with the entire 10-min exercise period marking the endpoint.

### Evaluation of autonomic activity, intensity and mind states

To minimize interference with biological rhythms, all tests were conducted between 8:00 a.m. and 12:00 p.m. in a controlled environment at 20°C–25°C.

Autonomic activity was assessed using heart rate variability (HRV) analysis. Raw RR interval data were initially collected using Polar tables (Polar Team2, Finland) and subsequently processed in Kubios software for moderate noise reduction. The researcher selected the smoothest 5-min RR interval values from the 10-min data during both quiet and Tai Chi periods for time and frequency domain metric calculations. The following time-domain indicators were analyzed: the square root of the mean squared differences of successive NN intervals (RMSSD), low frequency power (LF), high frequency power (HF), and the LF/HF ratio. LF and HF values were standardized as LF (nu) and HF (nu) to facilitate intergroup comparisons.

Exercise intensity (heart rate) data were collected using a Polar meter (Polar Team2, Finland). The final data were analyzed using the same segment as that used for heart rate variability assessment.

Respiratory indices were measured using a portable gas metabolism analyzer (MetaMax 3B, Germany). Respiratory parameters, including relative oxygen uptake, respiratory rate, tidal volume, and minute ventilation, were evaluated.

Mind activity during Tai Chi was assessed using the BFS Mood Scale administered before and after practice. This scale demonstrates high validity and has been extensively utilized to evaluate changes in mood states pre- and post-exercise. The BFS Mood Scale was developed by Zerssen in 1970 (31). The BFS Mood Scale serves as a mindfulness measurement tool grounded in the two-dimensional components of mindfulness (positivity and negativity) (32). The scale evaluates changes in the subject's mood, encouraging them to report their genuine feelings at that moment. It consists of eight subscales: Vitality, Pleasure, Thoughtfulness, Calmness, Anger, Excitability, Depression, and Inactivity. Each subscale contains five questions, yielding a total of 40 questions. Questions were randomly ordered, and respondents rated their experiences on a 5-point Likert scale, ranging from “not at all” to “completely”. The BFS Mood Scale is user-friendly, requiring approximately 5 min for completion. The Chinese version of the BFS Mood Scale was employed to ensure language consistency and facilitate participant completion. The BFS Mood Scale was translated into Chinese in 1997, followed by validity testing. Previous research has integrated the scale into Tai Chi studies for health promotion and assessed the mood states of college student populations (33).

### Statistical analysis

All data were analyzed statistically using SPSS 23. One-way ANOVA was employed to assess between-group variability of indicators during quiet and Tai Chi periods across different subject groups. Indicators failing to meet variance assumptions were transformed using natural logarithm (Ln) prior to analysis. Paired-samples *t*-tests were conducted to evaluate within-group differences for each index during quiet and Tai Chi periods. Statistical significance was set at *P* < 0.05.

## Results

### Characteristics of autonomic activity in quiet and Tai Chi states in different groups

Table 2 indicates that heart rate variability indices, specifically RMSSD and SDNN, differed significantly among the three groups of Tai Chi practitioners (*P* < 0.05). RMSSD and SDNN were significantly greater in the Traditional Chinese Medicine Students (TCMS) group compared to the Competitive Tai Chi Students (CTCS) and Economics and Management Students (EMS) groups. No significant differences were observed in HF (nu) and LF (nu) among the three groups of Tai Chi practitioners (*P* > 0.05). Notably, TCMS exhibited the highest HF (nu) and the lowest LF (nu) values. Comparisons between the quiet and Tai Chi states revealed significant changes across all three groups. Parasympathetic indicators, including RMSSD, SDNN, and HF (nu), significantly decreased during Tai Chi practice. Conversely, the sympathetic index LF (nu) increased significantly.

**Table 2:** Results of heart rate variability indexes in quiet and Tai Chi states in practitioners of different groups.

Index	Quiet state	Tai Chi state	P_1_	P_2_	P_3_
RMSSD, ms					
CTCS	37.70 ± 8.05	6.96 ± 4.91	0.000*	0.377	0.000*
TCMS	50.98 ± 16.67	12.04 ± 7.89			0.000*
EMS	25.43 ± 8.93	9.54 ± 7.92			0.000*
SDNN, ms					
CTCS	58.95 ± 9.81	11.06 ± 6.15	0.001*	0.264	0.000*
TCMS	65.71 ± 21.93	20.56 ± 13.25			0.001*
EMS	34.47 ± 11.88	14.84 ± 13.00			0.000*
HF (nu), %					
CTCS	54.33 ± 21.08	22.76 ± 10.53	0.146	0.417	0.001*
TCMS	74.13 ± 18.83	33.00 ± 24.43			0.005*
EMS	64.50 ± 18.11	24.82 ± 10.25			0.000*
LF (nu), %					
CTCS	45.51 ± 21.10	77.09 ± 10.12	0.149	0.417	0.001*
TCMS	25.76 ± 18.84	66.59 ± 24.94			0.005*
EMS	35.34 ± 18.22	74.86 ± 10.53			0.000*

P1 is the ANOVA result of the three groups of Tai Chi practitioners in the quiet state. P2 is the ANOVA result of the three groups of Tai Chi practitioners in the Tai Chi state. P3 is the paired *t*-test result of the quiet state vs. the Tai Chi state within the group. CTCS representing competitive Tai Chi students, TCMS representing Traditional Chinese medicine students, EMS representing Economics and management Students.

* Indicates a significant difference in results at *P* < 0.05.

### Characteristics of intensity in quiet and Tai Chi states in different groups

Table 3 demonstrates that heart rate indices did not differ significantly among the various groups of Tai Chi practitioners, either in the quiet state or during Tai Chi practice (*P* > 0.05). However, within each group, a significant increase in heart rate was observed when comparing the quiet state to the Tai Chi state (*P* < 0.05).

**Table 3:** Results of heart rate indexes in quiet and Tai Chi states in practitioners of different groups.

Index	Quiet state	Tai Chi state	P_1_	P_2_	P_3_
HR, beat/min					
CTCS	75.00 ± 6.82	130.00 ± 19.82	0.095	0.259	0.000*
TCMS	68.86 ± 5.62	127.63 ± 21.55			0.000*
EMS	76.78 ± 9.01	115.00 ± 17.95			0.000*

P1 is the ANOVA result of the three groups of Tai Chi practitioners in the quiet state. P2 is the ANOVA result of the three groups of Tai Chi practitioners in the Tai Chi state. P3 is the paired *t*-test result of the quiet state vs. the Tai Chi state within the group. CTCS representing competitive Tai Chi students, TCMS representing Traditional Chinese medicine students, EMS representing Economics and management Students.

* Indicates a significant difference in results at *P* < 0.05.

### Characteristics of respiration in quiet and Tai Chi states in different groups

Table 4 indicates that there were no significant differences in respiratory indices among the three groups of Tai Chi practitioners in the quiet state (*P* > 0.05). In the Tai Chi state, tidal volume was significantly higher in the CTCS group compared to both TCMS and EMS groups (*P* < 0.05), while other respiratory indicators showed no significant differences (*P* > 0.05). Comparisons of each group's quiet state with their Tai Chi state revealed significant increases in relative oxygen uptake, respiratory rate, tidal volume, and minute ventilation (*P* < 0.05).

**Table 4:** Results of respiratory indexes in quiet and Tai Chi states in practitioners of different groups.

Index	Quiet state	Tai Chi state	P_1_	P_2_	P_3_
Relative oxygen uptake, L/min					
CTCS	0.305 ± 0.051	1.236 ± 0.173	0.459	0.147	0.000*
TCMS	0.275 ± 0.040	1.141 ± 0.199			0.000*
EMS	0.297 ± 0.067	1.019 ± 0.237			0.000*
Respiratory rate, times/min					
CTCS	12.046 ± 3.373	20.432 ± 3.876	0.086	0.388	0.000*
TCMS	12.985 ± 3.500	21.847 ± 4.875			0.000*
EMS	15.622 ± 3.046	23.060 ± 5.669			0.000*
Tidal volume, L					
CTCS	0.804 ± 0.222	1.463 ± 0.267	0.051	0.005*	0.000*
TCMS	0.631 ± 0.123	1.241 ± 0.209			0.000*
EMS	0.621 ± 0.137	1.078 ± 0.163			0.000*
Minute ventilation, L/min					
CTCS	9.035 ± 1.792	28.951 ± 4.923	0.094	0.360	0.000*
TCMS	7.845 ± 1.314	26.471 ± 5.477			0.000*
EMS	9.233 ± 1.181	24.213 ± 5.926			0.000*

P1 is the ANOVA result of the three groups of Tai Chi practitioners in the quiet state. P2 is the ANOVA result of the three groups of Tai Chi practitioners in the Tai Chi state. P3 is the paired *t*-test result of the quiet state vs. the Tai Chi state within the group. CTCS representing competitive Tai Chi students, TCMS representing Traditional Chinese medicine students, EMS representing Economics and management Students.

* Indicates a significant difference in results at *P* < 0.05.

### Characteristics of mind in quiet states and immediately after Tai Chi in different groups

Table 5 illustrates that intergroup comparisons among the three Tai Chi practitioner groups showed no significant differences in mood indicators between the quiet state and immediately after Tai Chi practice (*P* > 0.05). Within the TCMS group, comparative analysis revealed significant decreases in thoughtfulness, anger, and inactivity following Tai Chi practice (*P* < 0.05), while other mood indicators remained unchanged (*P* > 0.05). In the EMS group, thoughtfulness significantly decreased post-Tai Chi practice (*P* < 0.05), while other values did not show significant changes (*P* > 0.05). No significant differences were observed in any mood indicators for the CTCS group (*P* > 0.05).

**Table 5:** List of changes in state of mind indices during quiet time and immediately after Tai Chi in different groups.

Index	Quiet State	Immediately after Tai Chi	P_1_	P_2_	P_3_
Vitality, score					
CTCS	19 ± 5	18 ± 4	0.426	0.566	0.378
TCMS	17 ± 4	18 ± 5			0.245
EMS	16 ± 3	16 ± 3			0.457
Pleasure, score					
CTCS	17 ± 4	18 ± 5	0.540	0.571	0.264
TCMS	19 ± 3	19 ± 5			1.000
EMS	17 ± 2	16 ± 3			0.366
Thoughtfulness, score					
CTCS	10 ± 5	10 ± 5	0.215	0.218	0.593
TCMS	10 ± 5	7 ± 2			0.035*
EMS	13 ± 3	9 ± 2			0.014*
Calmness, score					
CTCS	18 ± 4	18 ± 4	0.343	0.604	0.454
TCMS	18 ± 3	19 ± 5			0.445
EMS	16 ± 4	17 ± 3			0.591
Anger, score					
CTCS	7 ± 2	6 ± 1	0.985	0.564	0.322
TCMS	6 ± 2	5 ± 1			0.041*
EMS	7 ± 2	6 ± 1			0.496
Excitability, score					
CTCS	7 ± 4	7 ± 3	0.783	0.859	0.671
TCMS	8 ± 3	7 ± 2			0.232
EMS	8 ± 3	7 ± 3			0.299
Depressive, score					
CTCS	7 ± 3	6 ± 2	0.650	0.417	0.107
TCMS	6 ± 2	5 ± 1			0.068
EMS	7 ± 2	7 ± 2			0.565
Inactivity, score					
CTCS	8 ± 5	7 ± 4	0.845	0.721	0.103
TCMS	8 ± 3	6 ± 2			0.027*
EMS	8 ± 2	7 ± 2			0.158

P1 is the ANOVA result of the three groups of Tai Chi practitioners in the quiet state. P2 is the ANOVA result of the three groups of Tai Chi practitioners in the Tai Chi state. P3 is the paired *t*-test result of the quiet state vs. the Tai Chi state within the group. CTCS representing competitive Tai Chi students, TCMS representing Traditional Chinese medicine students, EMS representing Economics and management Students.

* Indicates a significant difference in results at *P* < 0.05.

## Discussion

This study explored the autonomic activity of Tai Chi practitioners from different professional backgrounds. TCMS exhibited significantly higher autonomic activity compared to CTCS and EMS in the quiet state. Following Tai Chi practice, the TCMS group demonstrated a tendency toward a calmer state of mind, although this was not significantly different from CTCS and EMS.

### Autonomic activity

In the quiet state, the time domain indexes RMSSD and SDNN of TCMS were significantly higher than the other two groups. Although not statistically significant, the frequency domain metrics HF (nu) and LF (nu) in TCMS exhibited a tendency toward significant differences. This suggests that time-domain indices may be more sensitive than frequency-domain indices. Previous studies have also concluded that the time-domain index RMSSD is more accurate in evaluating autonomic activity and recommended its use for monitoring (34). The combined analysis of time and frequency domain indices indicated that TCMS had higher autonomic activity than CTCS and EMS in the quiet state. This finding may be attributable to the professional background of TCMS practitioners. Chinese medicine theory serves as a foundational principle of Tai Chi, establishing a close relationship between the two. Within Chinese medicine, Tai Chi is categorized as a form of exercise therapy. Tai Chi practices are informed by Chinese medicine theories, aiming to regulate both mental and physical states while enhancing overall fitness. Long-term Tai Chi practice, integrated with Chinese medicine theory, may more effectively regulate autonomic function. CTCS practitioners focus on competitive Tai Chi, emphasizing the coordination of breathing and movement to enhance athletic performance. The high intensity and demands of competitive Tai Chi may overstimulate internal organs, potentially suppressing parasympathetic nerve activity. Autonomic activity is particularly sensitive to exercise intensity during physical activity (35). Within-group comparisons between exercise and quiet states revealed significant differences in autonomic activity indices across all three groups. However, no significant differences in autonomic activity were observed among the three groups during exercise. This suggests that the level of autonomic stimulation was consistent across all groups during Tai Chi practice.

### Intensity

Tai Chi is classified as an aerobic exercise. Tai Chi practice can induce autonomic excitation by stimulating both internal and external receptors (33). Sustained intensity during Tai Chi practice enhances autonomic activity (36). This study found no significant differences in heart rate indices among the three groups in both quiet and exercise states. This finding suggests that exercise intensity can elicit changes in autonomic activity. However, exercise intensity did not appear to be the primary factor influencing differences in autonomic activity among the three Tai Chi practitioner groups.

### Respiration

No significant differences were observed in relative oxygen uptake among the three groups during the quiet state. This indicates that baseline relative oxygen uptake was consistent across all three groups in the quiet state. Within-group comparisons revealed significantly higher relative oxygen uptake values during Tai Chi practice compared to the quiet state for all three groups. The distinctive breathing patterns associated with Tai Chi significantly enhance oxygen uptake (37). However, no significant differences were found in respiratory rate, tidal volume, and minute ventilation among the different Tai Chi practitioner groups in the quiet state. Within-group comparisons of data from quiet and exercise states revealed significant differences. Further analysis indicated that the CTCS group exhibited the lowest respiratory rate and the highest tidal volume in the quiet state. Long-term training may lead to beneficial adaptations in breathing patterns (38). If this adaptation is a key factor in increasing autonomic activity, especially parasympathetic activity in the quiet state, parasympathetic activity should be higher in CTCS than in TCMS. Contrary to this expectation, the present study found the opposite results. Respiratory rate, tidal volume, and minute ventilation were lower in TCMS compared to CTCS. Nevertheless, parasympathetic activity was significantly higher in TCMS than in CTCS. This suggests that the deep, prolonged, and slow breathing characteristic of Tai Chi may induce stress responses in the respiratory center and cardiovascular reflexes, leading to changes in autonomic activity (39). However, this was not the primary factor responsible for the differences in autonomic activity among the three groups. Therefore, the elevated parasympathetic activity observed in TCMS during the quiet state may not be solely attributable to Tai Chi breathing patterns.

### State of mind

Meditation plays a crucial role in promoting health (40). Research has shown that the meditative aspects of Tai Chi significantly reduce anxiety, depression, and stress (41). Neuroimaging studies indicate that Tai Chi meditation effectively stimulates various cortical regions, leading to adaptive changes (42). These changes may underlie the mechanisms through which Tai Chi enhances psychoemotional well-being, attentional transformation, and potentially delays aging (43). Increased activity in the left anterior cingulate cortex, medial prefrontal cortex, and medial temporal lobe is strongly linked to emotional processing (44). Bilateral hippocampal activity is associated with emotion regulation, stress response, and memory functions (45). Some researchers propose that Tai Chi may enhance inhibitory functions, as evidenced by increased prefrontal N2 and parieto-occipital P3 amplitudes (46).

Heart rate variability indices can reflect mood changes associated with autonomic activity (47). The results indicated no significant differences in the state of mind among the three groups during the quiet state. Within-group comparisons demonstrated significant reductions in thoughtfulness, anger, and inactivity in TCMS following exercise. Inactivity levels in EMS also decreased significantly. No significant differences were observed in any mood indicators for the CTCS group. This suggests that all three groups shared a similar baseline state of mind in the quiet state. Variability exists in the emotional experiences associated with Tai Chi practice. TCMS practitioners reported the highest levels of positive emotions and the most significant reductions in negative emotions during Tai Chi practice. However, immediately following Tai Chi practice, no significant differences were observed in mood indicators among the three groups. This may be related to the shorter duration of Tai Chi practice. It is plausible that the observed trends in TCMS would become more pronounced with an extended practice duration of 5–10 min. This trend may arise from the differing long-term focuses on techniques among the three groups. CTCS emphasizes the coordination of breathing patterns and movement performance in its Tai Chi practice. TCMS practice is guided by Traditional Chinese Medicine (TCM) theory and emphasizes intentional meditation. EMS practice centers on individual movements and their coherence. Consequently, this study concluded that the level of intentional meditation in TCMS practitioners is higher than that in CTCS and EMS practitioners. Intentional meditation in TCMS may more effectively stimulate activity in the left anterior cingulate cortex, medial prefrontal cortex, medial temporal lobe, and other emotion-regulating regions, leading to significant changes. This stimulation may alter signaling pathways, resulting in increased parasympathetic activity and decreased sympathetic activity (48). Such stimulation may induce long-term adaptive changes in relevant cortical areas and autonomic function (49). Consequently, TCMS practitioners tend to achieve a calmer state of mind following Tai Chi practice. Wei's study similarly concluded that Tai Chi breathing was not a primary factor in inducing adaptive changes in autonomic activity (50). The state of meditative relaxation is a key factor contributing to increased parasympathetic nerve activity (51). This perspective aligns with the findings of the current study. The meditative and intentional aspects of Tai Chi may play a crucial role in enhancing autonomic activity (52). This may explain the elevated parasympathetic activity observed in subjects during the quiet state, as well as the significant changes in their state of mind before and after exercise.

### Limitation

The sample size of this study could not be increased due to challenges in recruiting participants. This study is only a preliminary exploration of the characteristics of different Tai Chi practice groups and requires further validation through larger-scale experiments with larger sample sizes. In future studies, if funding and resources permit, the sample size can be expanded for validation, and the heart rate variability, respiratory indicators, and state of mind during the immediate recovery period after exercise can be further explored.

## Conclusion

Preliminary findings suggest that Tai Chi exercises, particularly those emphasizing mindfulness, may more effectively enhance parasympathetic activity while inhibiting sympathetic activity. Activity and inhibit sympathetic activity. It recommends the standing poles interventions for depression, insomnia, and other groups for more autonomic health benefits. The findings of this study provide evidence to support the development of clinical Tai Chi intervention programs for patients with chronic diseases.

## Data Availability

The raw data supporting the conclusions of this article will be made available by the authors, without undue reservation.
